# The association between visual impairment, educational outcomes, and mental health: insights from eyeglasses usage among junior high school students in rural China

**DOI:** 10.1038/s41598-024-72119-2

**Published:** 2024-10-16

**Authors:** Xiaodong Pang, Huan Wang, Yiwei Qian, Sabrina Zhu, Yuwei Adeline Hu, Scott Rozelle, Nathan Congdon, Jiting Jiang

**Affiliations:** 1 College of Education, Capital Normal University, Beijing, China; 2 Stanford Center on China’s Economy & Institutions (SCCEI), Freeman Spogli Institute for International Studies, Stanford University, Stanford, CA USA; 3 Research Institute of Economics and Management, Southwestern University of Finance and Economics, Sichuan, China; 4 Agricultural and Resource Economics, University of California, Davis, CA 95616 USA; 5 School of Medicine, Dentistry and Biomedical Sciences, Queen’s University Belfast, Belfast, Northern Ireland UK; 6 Orbis International, New York, NY USA

**Keywords:** Visual impairment, Educational outcomes, Mental health, Eyeglasses usage, Rural China, Junior high school children, Psychology, Human behaviour

## Abstract

This study examined the association between visual impairment, visual impairment corrected by wearing eyeglasses, academic performance, and mental health among junior high school students in rural China. Visual acuity assessments were conducted on 19,425 junior high school students by trained medical and research professionals to determine the presence of visual impairment. All sample students were surveyed with a questionnaire that asked about individual and family characteristics, eyeglasses ownership, and educational aspirations and included a standardized math test. Students then completed an official Chinese simplified version of the Strengths and Difficulties Questionnaire (SDQ) to assess their mental health multidimensionally. Among our sample, 37.2% of them suffered from visual impairment, but only 43.4% of the visually impaired students wore proper eyeglasses. Approximately 9.3% of students were categorized as high risk for mental health problems based on their SDQ score. A significant positive association existed between impaired vision and poorer mental health, and eyeglasses usage was associated with better mental health among visually impaired students. For students with better academic performance, eyeglasses usage associated with better mental health. Eyeglasses usage shows a positive association with higher education aspiration both for students with better and worse academic performance. The significant positive relationship between eyeglasses usage and mental health may guide future interventions and policies designed to improve student mental health by supplying them with eyeglasses.

## Introduction

Visual impairment (vision acuity < 6/12) is among the most common health problems worldwide, comprising half of all disabilities among young people^1^. Uncorrected refractive error is currently the leading cause of visual disability among school-aged children^2^. Although refractive error can be managed safely and inexpensively with properly fitted eyeglasses^3^, a significant number of children in need of refractive correction remain untreated^4–9^. For example, between 64% and 85% of refractive error cases in rural China remained uncorrected by eyeglasses in a population-based study^10^. Even in high-income countries, such as Australia, one out of every four children in need of eyeglasses do not have them^11^.

Visual impairment has been associated with mental illness such as depression and anxiety among children, with the strongest associations observed when refractive error remains uncorrected^7,12,13^. One possibility for this association is that students who struggle to see clearly in class may experience depression and anxiety symptoms due to learning difficulties^14^. Students with poor vision may also feel excluded by classmates without visual impairment who are able to participate more readily in classroom activities, leading to an increase in peer-related anxiety in visually impaired students^15,16^. It is important to note that children’s mental health issues are multidimensional and may affect them in different ways. These considerations call for a comprehensive assessment of children’s mental health and its relationship with visual impairment.

Despite increasingly strong evidence of an association, the precise mechanisms linking the use of eyeglasses to children’s mental health are yet to be fully understood. Nevertheless, it is evident that supplying eyeglasses for vision correction can enhance academic performance^4,17^. Moreover, improved educational outcomes from wearing eyeglasses could lead to better mental health^18^.

Visual impairment caused by uncorrected refractive error may be especially likely to exacerbate student mental health problems in countries with competitive educational systems and limited resources. These competitive educational systems appear to place even adolescents without vision impairment at higher risk of mental health problems^6,19^. Because of the potential synergy between academic stress, visual impairment, and mental health conditions such as anxiety and depression, studies in countries with documented high levels of student academic stress and uncorrected visual impairment are of particular importance.

Rural China is such a setting. Approximately half of the 5.9 million children worldwide with impaired vision due to uncorrected refractive error live in China^20^. In 2019, 54% of junior high school students in China were found to be myopic^21^. Despite this high prevalence, less than a quarter of students needing eyeglasses in rural and urban migrant Chinese settings own them^4–6,21^. In addition, even when free eyeglasses are provided to visually impaired children, many do not wear them regularly, due mainly to concerns that use of glasses might weaken their eyes^3,22^.

The aim of this paper is to address existing gaps in the literature and to determine the relationship between impaired vision, eyeglasses usage, mental health, and educational outcomes among junior high school students in rural China. We chose to study junior high school students because the prevalence of uncorrected refractive error and unmet need for eyeglasses peaks at this age, as does academic pressure as they prepare for the highly-competitive national high school entrance examination^5,23–25^. To achieve the study aim, we assessed visual acuity, eyeglasses wear, academic achievement, and mental health status in a population sample of 19,425 junior high school students in rural western China with data gathered in 2019. We had four objectives. First, we examined the prevalence of student visual impairment by trained medical and research professionals. Second, we examined the rates of student mental health problems according to their Strengths and Difficulties Questionnaire (SDQ) scores. Third, we investigated the associations between students’ visual impairment, visual impairment corrected by eyeglasses, and mental health. Fourth, we examined heterogeneous effects by analyzing how the use of eyeglasses among students with high or low academic performance influenced both their mental health and educational aspirations.

## Methods

Ethics approval for this study was granted by the Institutional Review Boards at Stanford University (Stanford, CA, USA), Queen’s University Belfast (Belfast, United Kingdom), and the Zhongshan Ophthalmic Center, Sun Yat-Sen University (Guangzhou, China). Permission was given by the Ningxia Board of Education and principals from the surveyed junior high schools, and informed consent was provided by at least one parent of all participating children. The principles of the Declaration of Helsinki were followed throughout the study.

### Study location and sampling

We drew our sample from an ongoing trial involving junior high school students in Ningxia, a landlocked, predominantly rural autonomous region in northwest China. In 2018, Ningxia has an area of 66,400 km^2^ and a population of 7.1 million, 63% of whom are ethnic Han and 36% of which belonging to China’s Muslim Hui minority. Ningxia’s per capita gross domestic product was $7517 in 2018, some 24% below the national average^26^. The initial survey was carried out in October 2019.

A two-step selection protocol was used to obtain a representative sample of all rural junior high school students in Ningxia. In the first step, the sample schools were selected from a list of all 273 junior high schools in Ningxia, acquired from local education bureaus in the six prefectures. To more closely focus on the rural population, students from the 122 schools in Yinchuan, the capital city of the autonomous region, were excluded from the sample. Schools with fewer than 40 children in 7th grade were also excluded due to logistical constraints. All other schools were included in the study, for a total of 124 participating institutions. In the second step, in each of the sampled junior high schools, at most two classes per grade were randomly selected from among all 7th and 8th grade classes (with children largely falling between the ages of 12 to 15 years). The final sample consisted of 20,375 students from 474 classes at 124 schools. A total of 19,425 students returned survey forms with complete data, forming our final analytical sample.

### Data collection

Data were collected by teams of trained enumerators using a survey instrument consisting of two components: a questionnaire and a visual acuity (VA) assessment.

#### Questionnaire survey

Enumerators distributed questionnaires to students to collect information about their demographic characteristics, academic performance, mental health. The demographic survey covered details such as student gender, ethnicity, rural residential status of the family, only child status, student grade, boarding at school, attendance of extracurricular classes, parental divorce, parental out-migration for work, grandparent(s) as the primary caregiver, parents’ age and years of schooling, household assets index, educational and career aspirations, and ownership of glasses. We also conducted a 30-min standardized mathematics test to assess student academic performance. The test was designed with input from local education experts to ensure coherence with China’s national curriculum. All scores were normalized by grade level.

An official Chinese simplified version of the Strengths and Difficulties Questionnaire (SDQ) screening instrument for children was used to determine students’ mental health status. The SDQ has a strong internal consistency (Cronbach’s alpha coefficient = 0.81) and high levels of reliability (Pearson’s correlation coefficient = 0.71)^27,28^. The questionnaire includes 20 items on behavioral strengths and difficulties, scored on a 3-point scale (0 = not true, 1 = somewhat true, and 2 = certainly true). Measured items are grouped into four subscales (0–10 points, with higher score indicating greater difficulties): emotional problems, conduct, hyperactivity/inattention, and peer relationships.

We use both SDQ score and SDQ abnormality to measure students’ mental health. The SDQ score, including SDQ total score, Internalizing SDQ score, and Externalizing SDQ score, was used in our data analysis. For SDQ abnormality, we scored the SDQ questionnaire according to the guidance provided on the SDQ official website; the abnormal score ranges are: 20–40 for total difficulties, 7–10 for emotional problems, 5–10 for conduct, 7–10 for hyperactivity, and 6–10 for peer problems^29^. We mainly used amalgamated scales that are sometimes used instead of the four separate difficulties subscales described above^30^. These alternative scales are called the ‘internalizing’ (comprising the emotional and peer problem subscales) and ‘externalizing’ subscales (comprising the conduct and hyperactivity subscales). Referencing Yu et al.^31^, we defined the student with abnormal internalizing problems if the students obtained abnormal scores on either emotional problems or peer problems subscales. We defined the student with abnormal externalizing problems if the students obtained abnormal scores on either conduct problems subscales or on hyperactivity subscales.

#### Visual acuity (VA) assessment

VA assessment was conducted at each school by an optometrist and a trained research assistant. For each student, each eye was tested individually at 4 m without refraction using Early Treatment Diabetic Retinopathy Tumbling-E study charts (Precision Vision, La Salle, IL) in an illuminated indoor space^4^. During the testing process, students moved from the 6/60 line to each successively lower line if they were able to identify the orientation of at least four out of five optotypes, to a maximum vision of 6/3. VA in an eye was identified as the lowest line for which the student was able to correctly determine at least four out of five optotypes. If the student could not read the 6/60 line, the chart was positioned at a distance of 1 m, and the resulting visual acuity was divided by 4. The assessment included cycloplegic automated refraction with subjective refinement to ascertain prescriptions for eyeglasses eligibility (the myopia cutoff is ≤  − 0.5 diopters [D]). The eye examination team was trained by Zhongshan Ophthalmic Center (ZOC) at Sun Yat-Sen University.

### Statistical analysis

First, we presented summary statistics encompassing student characteristics, mental health measured through SDQ, educational aspirations, and academic performance assessed by standardized math tests across four groups: the total sample, students with normal vision, students with vision impairment wearing glasses, and students with visual impairment without eyeglasses. We then performed t-tests to analyze disparities in demographics, mental health, educational aspirations, and academic performance between students with visual impairment wearing glasses and those with normal vision, as well as between students with visual impairment without glasses and those with normal vision. Second, we conducted several multivariate Ordinary Least Squares (OLS) regressions to examine associations between student vision, eyeglass usage, and mental health after controlling for student characteristics and math tests. Third, we divided our sample into a high academic performance group, defined by standardized math tests scores greater than or equal to the median, and a low academic performance group, defined by standardized math test scores lower than the median. We then ran multivariate OLS regressions to explore the associations between student eyeglasses usage and mental health within the high and low academic performance groups, controlling for student characteristics and math scores. Fourth, we ran multivariate OLS regressions to examine associations between student eyeglasses usage and educational aspiration among the high/low academic performance groups, respectively, after controlling for student characteristics and math scores. All statistical analyses were performed using Stata 17.0 (Stata Corp LLC, Texas, USA).

## Results

Table 1 provides summary statistics for the sampled students. Out of 19,425 students, 37.2% (N = 7221) had poor vision, 56.6% (N = 4088) of whom did not wear glasses. Additionally, 48.6% (N = 9440) were female, 64.9% (N = 12,606) belonged to the Hui ethnic minority group, 93.6% (N = 18,181) resided in rural areas, 6.8% (N = 1320) were only children, and 34.8% (N = 6760) had at least one parent who had migrated for work in the past 6 months and did not live at home. A 9.7% divorce rate among parents was observed. Fathers had a mean age of 41.3 years and an average of 7.08 years of schooling, while mothers had a lower mean age of 38.67 and fewer years of schooling, with a mean of 5.13 years. Among the children, 35.4% (N = 6876) reported boarding at school, and 11.3% (N = 2195) attended extracurricular classes. As indicated in Table 1, there was a significantly higher prevalence of girls among students with visual impairment with/without glasses (62.5%/55.4%) compared to those with normal vision (42.7%) (p < 0.001).

**Table 1:** Summary statistics of sample students and their family characteristics.

Variable	Full sample	By visual impairment			Differences	
	N = 19,425	Normal vision N = 12,204	Visual impairment with glasses N = 3133	Visual impairment without glasses N = 4088	Visual impairment with glasses vs normal vision	Visual impairment without glasses vs normal vision
	(1)	(2)	(3)	(4)	(3)–(2)	(4)–(2)
	Mean (SD)				Coefficient (P-value)	
Girl (1 = yes)	0.486 (0.499)	0.427 (0.494)	0.625 (0.484)	0.554 (0.497)	0.197 (< 0.001)	0.127 (< 0.001)
Hui ethnicity (1 = yes)	0.649 (0.477)	0.678 (0.466)	0.510 (0.499)	0.670 (0.470)	− 0.169 (< 0.001)	− 0.009 (0.299)
Rural residence (1 = yes)	0.936 (0.243)	0.940 (0.237)	0.920 (0.270)	0.939 (0.237)	− 0.019 (< 0.001)	− 0.000 (0.964)
Only child (1 = yes)	0.068 (0.251)	0.069 (0.254)	0.073 (0.260)	0.059 (0.236)	0.003 (0.501)	− 0.010 (0.028)
Grade (1 = seven)	0.495 (0.499)	0.523 (0.499)	0.390 (0.488)	0.489 (0.499)	− 0.133 (< 0.001)	− 0.035 (< 0.001)
Boarding at school (1 = yes)	0.354 (0.478)	0.345 (0.475)	0.360 (0.480)	0.376 (0.484)	0.015 (0.127)	0.031 (< 0.001)
Attending extracurricular classes (1 = yes)	0.113 (0.317)	0.114 (0.318)	0.124 (0.330)	0.103 (0.305)	0.010 (0.113)	− 0.010 (0.069)
Parents divorced (1 = yes)	0.097 (0.296)	0.100 (0.300)	0.082 (0.275)	0.099 (0.299)	− 0.018 (< 0.001)	− 0.001 (0.857)
Either parent migrated (1 = yes)	0.348 (0.476)	0.341 (0.474)	0.371 (0.483)	0.353 (0.478)	0.029 (< 0.001)	0.012 (0.173)
Grandparent as primary caregiver (1 = yes)	0.118 (0.323)	0.117 (0.322)	0.112 (0.316)	0.124 (0.329)	− 0.005 (0.437)	0.006 (0.285)
Father’s age (years)	41.343 (5.314)	41.343 (5.350)	41.231 (5.156)	41.431 (5.323)	− 0.112 (0.292)	0.088 (0.360)
Mother’s age (years)	38.674 (5.030)	38.653 (5.074)	38.755 (4.901)	38.677 (4.995)	0.103 (0.309)	0.025 (0.786)
Father’s education (years)	7.083 (3.202)	7.006 (3.221)	7.554 (2.977)	6.950 (3.282)	0.548 (< 0.001)	− 0.056 (0.339)
Mother’s education (years)	5.137 (4.015)	5.091 (3.992)	5.623 (4.048)	4.901 (4.026)	0.532 (< 0.001)	− 0.190 (< 0.009)
Household assets (1 = bottom third of sample)	0.333 (0.471)	0.336 (0.472)	0.273 (0.445)	0.368 (0.482)	− 0.063 (< 0.001)	0.032 (< 0.001)

Source: Authors’ survey.

Tables 2 presents summary statistics of student mental health, educational aspiration, and academic performance. On average, students obtained scores of 12.6, 6.4, and 6.1 on the Strengths and Difficulties Questionnaire (SDQ) for total scores, internalizing scores, and externalizing scores, respectively. Additionally, 9.0%, 15.1%, and 17.3% of students exhibited abnormal scores in SDQ total, internalizing, and externalizing categories, respectively. Students with visual impairment wearing glasses were 0.35 (p < 0.001), 0.27 (p < 0.001), 0.01 (p = 0.006), and 0.03 (p < 0.001) percentage points less likely to experience mental health problems in terms of their SDQ total score, externalizing score, SDQ prevalence of abnormal scores, externalizing abnormality, and externalizing abnormality than students with normal vision. Meanwhile, students with visual impairment without glasses were 0.27 (p < 0.001) and 0.23 (p < 0.001) percentage points more likely to have mental health problems in terms of their SDQ total score and externalizing score than students with normal vision.

**Table 2:** Distribution of mental health, education aspiration, and academic performance of sample students.

Variable	Full sample	By visual impairment			Differences	
	N = 19,425	Normal vision N = 12,204	Visual impairment with glasses N = 3133	Visual impairment without glasses N = 4088	Visual impairment with glasses vs normal vision	Visual impairment without glasses vs normal vision
	(1)	(2)	(3)	(4)	(3)–(2)	(4)–(2)
	Mean (SD)				Coefficient (P-value)	
Panel A: Student mental health						
Strengths and Difficulties Questionnaire (SDQ) total scores	12.605 (4.915)	12.604 (4.950)	12.253 (4.769)	12.876 (4.901)	− 0.351 (< 0.001)	0.272 (< 0.001)
Internalizing score	6.453 (2.980)	6.416 (2.983)	6.344 (2.944)	6.648 (2.991)	− 0.072 (0.225)	0.232 (< 0.001)
Externalizing score	6.151 (2.922)	6.188 (2.939)	5.909 (2.847)	6.227 (2.917)	− 0.279 (< 0.001)	0.040 (0.454)
SDQ prevalence of abnormal scores	0.093 (0.290)	0.094 (0.292)	0.078 (0.268)	0.102 (0.303)	− 0.016 (0.006)	0.009 (0.107)
Internalizing SDQ abnormality	0.151 (0.358)	0.150 (0.357)	0.138 (0.345)	0.161 (0.368)	− 0.012 (0.087)	0.011 (0.084)
Externalizing SDQ abnormality	0.173 (0.378)	0.178 (0.383)	0.146 (0.353)	0.178 (0.382)	− 0.032 (< 0.001)	− 0.001 (0.937)
Panel B: Student education aspiration						
Very likely to take high school entrance exam	0.728 (0.444)	0.709 (0.454)	0.802 (0.398)	0.727 (0.445)	0.093 (< 0.001)	0.018 (0.024)
Most likely to attend academic high school after junior high school	0.734 (0.442)	0.714 (0.451)	0.807 (0.394)	0.733 (0.442)	0.092 (< 0.001)	0.019 (0.020)
Most likely to attend vocation high school after graduation	0.208 (0.405)	0.221 (0.415)	0.155 (0.362)	0.208 (0.406)	− 0.065 (< 0.001)	− 0.013 (0.086)
Most likely to work after junior high school	0.058 (0.234)	0.063 (0.244)	0.037 (0.188)	0.057 (0.233)	− 0.027 (< 0.001)	− 0.006 (0.169)
Panel C: Student standardized test score						
Math test scores, (standardized)	0.006 (0.995)	− 0.037 (0.989)	0.261 (0.980)	− 0.054 (0.992)	0.299 (< 0.001)	− 0.017 (0.337)

Source: Authors’ survey.

The majority (N = 14,141, 72.8%) of students believed that they were very likely to take the high school entrance exam. Additionally, 73.4% of students expected to attend academic high school, 20.8% students expected to attend vocational high school (i.e., vocational education and training), and 5.8% of students expected to enter the labor market after graduation from junior high school. Students with visual impairment who wore glasses were 0.093 (p < 0.001) and 0.092 (p < 0.001) percentage points more likely to take the high school entrance exam and attend academic high school after graduation compared to students with normal vision. Surprisingly, students with visual impairment without glasses followed a similar pattern, with a higher likelihood of taking the high school entrance exam and attending academic high school after graduation compared to students with normal vision.

The large share of junior high school students who suffer from visual impairment and poor mental health status, as shown above, leads us to consider two important yet understudied questions: To what extent does visual impairment associate with mental health? What is the role of eyeglasses in mental health for students with visual impairment?

We began by examining the association between visual impairment and mental health in the overall sample. Table 3 panel A shows the results of our multivariate regression when student and family characteristics were held constant (as described earlier). We found significant associations between visual impairment and higher SDQ scores and, thus, poorer mental health. Specifically, we found that students with visual impairment scored 0.148 and 0.105 higher in their SDQ total score and internalizing score compared to students with normal vision. Second, we examined the associations between eyeglasses usage and mental health. The results of the analysis are presented in Table 3 Panel B. We found that the adverse impact of poor vision on mental health diminished when students with poor vision wore eyeglasses. Specifically, there were no significant differences in mental health between students with visual impairment wearing eyeglasses and those with normal vision. We further found that the negative impact of poor vision on mental health was more pronounced for students without eyeglasses. Specifically, students with visual impairment without eyeglasses scored 0.23 and 0.15 higher on their SDQ total score and internalizing score than students with normal vision. Third, we narrowed our sample to only those students with visual impairment to scrutinize the role of eyeglasses on mental health. The outcomes are presented in Table 3 Panel C, revealing a significant association between eyeglasses usage and improved mental health.

**Table 3:** Associations between student vision, eyeglasses usage and mental health.

	SDQ scores			SDQ abnormality		
	SDQ Total score	Internalizing SDQ score	Externalizing SDQ score	SDQ total abnormality	Internalizing SDQ abnormality	Externalizing SDQ abnormality
	(1)	(2)	(3)	(4)	(5)	(6)
Panel A: Full sample						
Normal vision as comparison						
Visual impairment (1 = yes)	0.148** (0.072)	0.105** (0.044)	0.044 (0.043)	0.004 (0.004)	0.002 (0.005)	0.002 (0.006)
Control variables	Yes	Yes	Yes	Yes	Yes	Yes
No. of observations	19,425	19,425	19,425	19,425	19,425	19,425
R^2^	0.060	0.045	0.054	0.022	0.016	0.031
Panel B: Full sample						
Normal vision as comparison						
Visual impairment with eyeglasses (1 = yes)	0.034 (0.098)	0.035 (0.060)	− 0.001 (0.059)	− 0.002 (0.006)	− 0.003 (0.007)	− 0.002 (0.008)
Visual impairment without eyeglasses (1 = yes)	0.230*** (0.087)	0.155*** (0.053)	0.075 (0.052)	0.008 (0.005)	0.006 (0.006)	0.005 (0.007)
Control variables	Yes	Yes	Yes	Yes	Yes	Yes
No. of observations	19,425	19,425	19,425	19,425	19,425	19,425
R^2^	0.075	0.052	0.051	0.027	0.019	0.038
Panel C: Among visual impairment students						
Visual impairment without eyeglasses as comparison						
Visual impairment with eyeglasses (1 = yes)	− 0.203* (0.116)	− 0.128* (0.072)	− 0.075 (0.069)	− 0.011 (0.007)	− 0.009 (0.009)	− 0.012 (0.009)
Control variables	Yes	Yes	Yes	Yes	Yes	Yes
No. of observations	7221	7221	7221	7221	7221	7221
R^2^	0.061	0.045	0.054	0.022	0.018	0.026
Avg Dep Var	12.605	6.453	6.151	0.093	0.151	0.173

Source: Authors’ survey. Standard errors in parentheses. *, **, and *** indicate significance at the 10%, 5%, and 1% levels of the coefficient.

We further examined the heterogeneous treatment effect of relationships between eyeglasses usage and mental health across high/low academic performance groups. The results in Table 4 reveal that eyeglasses usage may have protected mental health for students with high academic performance, but not for the students with low academic performance. Specifically, results in Table 4 Panel A show that among high academic performance students who wore eyeglasses, they scored lower in all SDQ measures except for internalizing SDQ abnormality compared to students with similar academic performance with visual impairment who did not wear eyeglasses. The result in Table 4 Panel B revealed that for the low academic performance students, there were no significant relationships between eyeglasses usage and mental health.

**Table 4:** Associations between student eyeglasses usage and mental health among students with poor vision, by academic performance.

	SDQ scores			SDQ abnormality		
	SDQ Total score	Internalizing SDQ score	Externalizing SDQ score	SDQ total abnormality	Internalizing SDQ abnormality	Externalizing SDQ abnormality
	(1)	(2)	(3)	(4)	(5)	(6)
Panel A: Standardized math test score above the median						
Visual impairment with eyeglasses (1 = yes)	− 0.436*** (0.158)	− 0.205** (0.098)	− 0.231** (0.095)	− 0.023*** (0.008)	− 0.017 (0.011)	− 0.028** (0.011)
Control variables	Yes	Yes	Yes	Yes	Yes	Yes
No. of observations	3696	3696	3696	3696	3696	3696
R^2^	0.023	0.027	0.019	0.010	0.009	0.008
Panel B: Standardized math test score below the median						
Visual impairment with eyeglasses (1 = yes)	− 0.092 (0.172)	− 0.105 (0.106)	0.014 (0.102)	− 0.003 (0.012)	− 0.006 (0.014)	0.001 (0.014)
Control variables	Yes	Yes	Yes	Yes	Yes	Yes
No. of observations	3525	3525	3525	3525	3525	3525
R^2^	0.025	0.027	0.026	0.017	0.013	0.018
Avg Dep Var	12.605	6.453	6.151	0.093	0.151	0.173

Source: Authors’ survey. Standard errors in parentheses. *, **, and *** indicate significance at the 10%, 5%, and 1% levels of the coefficient.

In Table 5, we present the results for examining the role of eyeglasses in raising or lowering educational aspirations among high/low academic performance groups. The results in Table 5 Panel A show that students with high academic performance who wore glasses had significantly improved educational aspirations compared to students with similar academic performance who did not wear glasses. This relationship pattern persists in students with low levels of academic performance, with the improved effect even stronger. Specifically, Table 5 Panel B shows that students with low academic performance who wore eyeglasses were associated with higher educational aspirations, such as believing that they were very likely to take the high school entrance exam, very likely to attend an academic high school after graduation, and less likely to enter the labor market after junior high school graduation.

**Table 5:** Associations between eyeglasses usage and educational aspirations among students with poor vision, by academic performance.

	Very likely to take high school entrance exam	Most likely to attend academic high school after graduation	Most likely to work after graduation
	(1)	(2)	(4)
Panel A: Standardized math test score above the median			
Visual impairment with eyeglasses (1 = yes)	0.019* (0.012)	0.022* (0.012)	− 0.002 (0.005)
Control variables	Yes	Yes	Yes
No. of observations	3696	3696	3696
R^2^	0.026	0.024	0.013
Panel B: Standardized math test score below the median			
Visual impairment with eyeglasses (1 = yes)	0.052*** (0.017)	0.050*** (0.017)	− 0.017* (0.009)
Controls variables	Yes	Yes	Yes
No. of observations	3525	3525	3525
R^2^	0.020	0.017	0.024
Avg Dep Var	0.728	0.733	0.058

Source: Authors’ survey. Standard errors in parentheses. *, **, and *** indicate significance at the 10%, 5%, and 1% levels of the coefficient.

## Discussion

We studied the multidimensional associations between visual impairment, eyeglasses use, and mental health among junior high school students in rural China. Using data from 19,425 students from 124 schools in the rural Ningxia region of northwest China, we found that 9.3% to 17.3% of these students were categorized as SDQ abnormal, and 37.2% had visual impairment. After studying associations, first, we found that students with visual impairment were more likely to have poorer mental health in terms of their SDQ total score and internalizing SDQ score. In addition, students with visual impairment who did not wear eyeglasses had significantly poorer mental health in terms of their SDQ total score and internalizing SDQ score compared to students with normal vision. Third, eyeglasses usage was significantly associated with improved mental health for the students with higher academic performance, but not for the students with lower academic performance. Finally, eyeglasses usage was significantly associated with higher educational aspirations for both high and low academic performance students.

The rural students in our study exhibited a high prevalence of mental health problems and had elevated rates of uncorrected visual impairment. The mean SDQ total difficulties score in this study was 12.60. In comparison, more developed nations, such as the United States, reported a mean score of 7.10^32^, and in a more urban region like Wuhan, China, the mean score was 11.24^33^. In our study, 37.2% of the sampled students had poor vision, a rate higher than the global average of 7.26%^34^. Moreover, a significant majority (56.6%) of the rural students with poor vision did not use eyeglasses. This trend mirrors findings from a previous study in the rural Chaoshan region of China, where two-thirds of students with visual impairment did not use eyeglasses^6^. The notable disparity in eyeglasses usage between rural and urban areas is evident, as 75% of urban Chinese students have visual impairment, yet a substantial majority (65.9%) own and use eyeglasses^5,21,44^.

The positive association that we found between visual impairment and student mental health problems supports the existing literature. Students in our study with visual impairment were significantly more likely to have poor mental health as measured by SDQ. These results agree with findings from a previous study conducted in rural China that sampled primary school students and found that visual impairment could have negative impacts on their academic achievement and mental health^13,17^. In the competitive education system in rural China, poor vision may discourage students from academically achieving, leading to feelings of hopelessness or defeat^35^.

Leveraging the multidimensional framework of the SDQ, our study provided deeper insights into specific aspects of student mental health, specifically internalizing or externalizing problems as measured by the SDQ subscales, and their associations with visual impairment compared to prior research. Our results highlighted a particularly significant association between visual impairment and internalizing problems. This finding suggests that visually impaired students may encounter heightened emotional conflicts related to self-image and peer relationships, potentially leading to challenges in forming friendships or facing bullying in class^14,36,37^. Our results align with existing literature suggesting that students with vision impairment are more likely to be bully-victims rather than bullies^13,38^. Moreover, literature on student bullying has shown that internalizing problems typically occur among victims, while externalizing problems are more common among bullies^39,40^. Therefore, we believe that because students with visual impairment are more susceptible to being bullied, they have higher internalizing problems.

Moreover, although wearing eyeglasses can safely and easily correct visual impairment, we did not observe significant differences in mental health between visually impaired students who wore eyeglasses and students with normal vision. This lack of association could be attributed to the potential negative mental health impacts associated with wearing eyeglasses, such as peer bullying, which may outweigh positive benefits^6,7,41^. However, our examination also revealed that students with visual impairment who did not wear eyeglasses exhibited significantly worse mental health than students with normal vision. Taken together, these findings suggest that eyeglasses are crucial for students with uncorrected visual impairment, and that their mental health may significantly decline if they do not wear eyeglasses. However, once visual impairment is corrected with eyeglasses, their mental health aligns with that of students with normal vision.

Heterogeneous effects impacting this association may also exist; for instance, specific groups of students could experience a strong positive impact from wearing eyeglasses and endure fewer negative effects, resulting in an overall positive net effect for these particular student groups^13^. As the students in our study navigate a highly competitive education system, their academic performance becomes a pivotal factor influencing various aspects of their school experience, including the decision to wear eyeglasses. In our pursuit of understanding heterogeneous effects, we investigated the association between eyeglasses usage and mental health among students with high and low academic performance. After categorizing junior high school students based on their standardized math test scores, we observed a significant association between eyeglasses usage and improved mental health for students with higher math scores. Specifically, high academic achievers who wore eyeglasses scored lower in all SDQ measures except for internalizing abnormality. A plausible explanation for these findings lies in the intense academic competition within rural China’s educational system, where students face substantial pressure to excel academically. In this context, students with higher academic proficiency may recognize that wearing eyeglasses enhances their learning capabilities, enabling them to achieve more and fostering confidence, thereby positively impacting their mental health^35,42^.

On the contrary, students with lower academic proficiency did not experience a significant change to their mental health after wearing eyeglasses. This result diverges from a prior study conducted in China, which identified a notable adverse impact on the mental health of visually impaired students with low academic performance after they started wearing eyeglasses^13^. This finding was attributed to prevalent social norms in China. According to a 2013 study, wearing eyeglasses is often associated with being perceived as more studious. Consequently, when low-performing students started wearing eyeglasses, they faced bullying from both peers and teachers because they were not perceived as studious but were now wearing eyeglasses^13^. The current study, conducted with 2019 data gathered in rural China, did not find a significant association between eyeglasses usage and poor mental health among students with low academic performance. This suggests a potential shift in social norms regarding eyeglasses usage in rural China, potentially indicating that knowledge about vision health has improved among rural residents.

Finally, we explored the relationship between eyeglasses usage and educational aspirations. First, we observed that wearing eyeglasses is associated with increased educational aspirations for both high and low academic performance students, possibly due to improved mental health boosting their confidence in learning^43^. Second, there is a heterogeneous effect, with the impact of eyeglasses on educational aspirations being over two times stronger for low academic performance students. The lower the academic performance, the more pronounced the positive effect of improved educational aspirations from wearing eyeglasses. Third, there is a significantly negative relationship between eyeglasses usage and the aspiration to work after graduation among low academic performance students, but not for high academic performance students. This may be explained by the persistence of high academic performance students in pursuing high school admission regardless of eyeglasses usage, whereas for low academic performance students in rural China, wearing eyeglasses significantly and strongly increases their confidence in attending academic high school after graduation, expanding their decision-making options and allowing them to choose not to join the labor market after graduation, a choice they might not have considered without the positive effects of eyeglasses.

## Conclusion

This study has several strengths. First, our study advances the literature on visual impairment, eyeglasses usage, student mental health, and student educational aspiration in the context of a developing country. Second, our utilization of data collected in 2019, coupled with a substantial sample size of 19,425 junior high school students, enabled a thorough and updated exploration of the relationships between visual impairment, visual impairment corrected by wearing eyeglasses, and mental health, capturing relevant associations in rural China and allowing for robust conclusions. Third, using subscales of the SDQ, we were able to identify more nuanced and specific mental health impacts of visual impairment and eyeglasses usage.

Several limitations should be acknowledged when considering our results. First, although the sample size was large, caution must be used in extrapolating the results across all rural areas of China or beyond. Second, it is advisable to conduct longitudinal research to establish causality between student mental health, visual impairment, and eyeglasses usage.

Despite these limitations, our study yields crucial insights into the ramifications of uncorrected visual impairment on child mental health development and underscores the necessity for targeted research and interventions aimed at bolstering the mental health of children who with visual impairment could be easily solved by distributing eyeglasses with low price that live in low-income rural settings. Policymakers should pay closer attention to the potential human capital loss and social costs of rural students’ vision impairment unadjusted borne by this already vulnerable subset of students. Urgent action seems to be called for to design interventions and support programs for uncorrected myopia of vulnerable students in low-income rural settings.

## Data Availability

The dataset used and/or analyzed during the current study are available from the corresponding author on reasonable request.
